# Treatment with Cyclic AMP Activators Reduces Glioblastoma Growth and Invasion as Assessed by Two-Photon Microscopy

**DOI:** 10.3390/cells10030556

**Published:** 2021-03-04

**Authors:** Krista Minéia Wartchow, Benjamin Schmid, Philipp Tripal, Andreas Stadlbauer, Michael Buchfelder, Carlos-Alberto Gonçalves, Andrea Kleindienst

**Affiliations:** 1 Department of Neurosurgery, Friedrich-Alexander University, 91054 Erlangen, Germany; kristawartchow@hotmail.com (K.M.W.); Andreas.Stadlbauer@uk-erlangen.de (A.S.); Michael.Buchfelder@uk-erlangen.de (M.B.); 2 Department of Biochemistry, Federal University of Rio Grande do Sul, 90035-003 Porto Alegre, Brazil; casg@ufrgs.br; 3 Institute of Optical Imaging Erlangen, 91054 Erlangen, Germany; benjamin.schmid@fau.de (B.S.); philipp.tripal@fau.de (P.T.)

**Keywords:** glioblastoma, treatment, Warburg effect, oxidative phosphorylation, 2-photon-microscopy

## Abstract

(1) Background: Despite progress in surgery and radio-chemotherapy of glioblastoma (GB), the prognosis remains very poor. GB cells exhibit a preference for hypoxia to maintain their tumor-forming capacity. Enhancing oxidative phosphorylation—known as the anti-Warburg effect—with cyclic AMP activators has been demonstrated to drive GB cells from proliferation to differentiation thereby reducing tumor growth in a cell culture approach. Here we re-evaluate this treatment in a more clinically relevant model. (2) Methods: The effect of treatment with dibutyryl cyclic AMP (dbcAMP, 1 mM) and the cAMP activator forskolin (50µM) was assessed in a GB cell line (U87GFP+, 10^4^ cells) co-cultured with mouse organotypic brain slices providing architecture and biochemical properties of normal brain tissue. Cell viability was determined by propidium-iodide, and gross metabolic effects were excluded in the extracellular medium. Tumor growth was quantified in terms of area, volume, and invasion at the start of culture, 48 h, 7 days, and 14 days after treatment. (3) Results: The tumor area was significantly reduced following dbcAMP or forskolin treatment (F_2,249_ = 5.968, *p* = 0.0029). 3D volumetric quantification utilizing two-photon fluorescence microscopy revealed that the treated tumors maintained a spheric shape while the untreated controls exhibited the GB typical invasive growth pattern. (4) Conclusions: Our data demonstrate that treatment with a cAMP analog/activator reduces GB growth and invasion.

## 1. Introduction

Glioblastoma (GB) has the highest prevalence and mortality among primary brain tumors [1], and treatment options consisting of surgery, radiotherapy, and chemotherapy have not substantially improved the poor prognosis [2,3]. Strategies to change the devastating prognosis of GB patients are therefore urgently required.

The Warburg effect describes the preference of cancer cells for glycolysis, even if the capacity for mitochondrial oxidative phosphorylation (OXPHOS) exists [4]. This effect was long misinterpreted as evidence for damage to respiration, but it is now recognized as a driving force in tumorigenesis [5]. While OXPHOS allows the generation of up to 36 mol ATP per mol glucose, glycolysis results just in 2 mol ATP on the benefit of 6 carbon molecules ready to enter cell replication essential for tumor proliferation [6].

Utilizing two established glioma cell lines—DBTRG-05MG and U87—originating from GB patients, Xing et al. demonstrated that the metabolic shift from glycolysis back to OXHPOS induced by cyclic AMP (cAMP) activators, known as the “anti-Warburg effect”, reduced tumor proliferation and facilitated astroglial differentiation [7]. Differentiation therapy, which is mechanistically different from most therapies aiming to kill cancer cells, has demonstrated significant clinical benefits in hematologic malignancies [8] and results in a complete remission of 90% [9]. However, these effects have never been achieved in GB.

Cyclic AMP and its downstream signals are intimately involved in regulating metabolic pathways, cell growth, and the cell cycle of the mammalian cell [10]. In several studies, the importance of cAMP signaling in glioma has been highlighted. It has been reported that cAMP suppression promotes gliomagenesis in a mouse model [11] and that cAMP elevation suppresses brain tumor growth in vitro and in vivo [12].

Transplantation of isolated or cultured GB cells into the adult rodent brain is the ‘gold standard’ to test treatment efficacy. However, these experiments are non-trivial and they do not enable direct monitoring of cell behavior, such as metabolic status, tumor growth, and invasion, or responses to treatment. Organotypic brain slice cultures (OBSC) overcome some of these difficulties as they provide access to brain tissue architecture in an intact biological environment thereby enabling the direct observation of treatment effects [13]. Micro-dissected brain slices are cultured above a semipermeable membrane in a cell culture insert and exposed to a serum-containing medium from below. Organotypic brain slices co-cultured with GB cells have been used to explore tumor–host cell interactions [14].

The evaluation of glioma growth, infiltration, or migration as well as the specific response to drug treatment has been performed ex vivo applying immunofluorescence [15]. While confocal laser scanning microscopy allows quantification of three-dimensional tumor growth co-cultured with organotypic brain slices in vivo [16], the depth of penetration is limited to 50–100 µm due to the scattering of light by tissue. However, organotypic brain slice thickness ranges around 300 µm and the diameter of the transplanted tumor spheres around 200 µm. Unlike confocal microscopy, excitation by two-photon (excitation > 700 nm) enables photons to penetrate tissue with a thickness of up to 1 mm, and highly sensitive confocal detection of emission signals can be detected without a pinhole at the depth of up to 1 mm.

To the best of our knowledge, we are the first to provide evidence for the efficacy of cAMP activators in the treatment of GB as assessed longitudinally by quantitative 3D microscopy. Continuing pure cell culture experiments [7], we demonstrate in an OBSC model preserving brain tumor interactions, that utilizing the “anti-Warburg effect”, i.e. the metabolic shift from glycolysis back to OXHPOS induced by cAMP activators, reduces the tumor-forming and invasive capacity and provides a differentiation therapy in GB. Two-photon fluorescence microscopy allows a valuable quantification and visualization of tumor structure, morphology, and three-dimensional growth within our OBSC model.

## 2. Materials and Methods

All animal experiments were approved by the Institutional Animal Care and Use Committee (No. TS-6/14) and in accordance with the National Research Council’s guide for the care and use of laboratory animals.

### 2.1. Organotypic Brain Slice Cultures

Organotypic brain slice cultures of 8-day-old C57BL/6 mice were prepared and maintained according to an established protocol [17]. Animal brains were removed and kept under ice-cold conditions. The cerebellum was removed. The remaining brain was embedded in 5% low melting agarose and cut into 350 µm thick coronal slices using a vibratome (Leica VT1000S, Bensheim, Germany). The brain slices were placed onto cell culture inserts (pore size 0.4 µm; Greiner BioOne, Frickenhausen, Germany) and subsequently transferred into six-well culture dishes (GreinerBioOne) containing 1.2 mL culture medium (MEM-HBSS, 2:1, 25% horse serum, 2% L-glutamine, 2.64 mg/mL glucose, 100 U/mL penicillin, 0.1 mg/mL streptomycin, 10 µg/mL insulin-transferrin-sodium selenite supplement, and 0.8 µg/mL vitamin C–pH was adjusted to 7.4 with 37% HCl and the medium was stored at 4 °C for up to 2 weeks). The slices were cultured in a humidified atmosphere (35 °C, 5% CO_2_) for up to 21 days. The medium was changed on the day following OBSC preparation and thereafter every second day. At the end of the experiments, the brain slice thickness was reduced to 100 μm, as expected for an organotypic culture.

### 2.2. U87 Glioblastoma Cell Line Culture and Transplantation

The green fluorescent protein (GFP)-transfected human GB cell line U87 (U87GFP+) was obtained from ATCC/LGC (Wesel, Germany). Cells were maintained at a temperature of 37 °C in an atmosphere of 5% CO_2_/95% air with Dulbecco’s modified Eagle’s medium (DMEM; Biochrom, Berlin, Germany) supplemented by 10% fetal bovine serum (FBS, Biochrom) and 1% penicillin/streptomycin (Biochrom). Cells were detached from the culture flasks using 0.05% trypsin/ethylenediaminetetraacetic acid (EDTA) before tumor transplantation.

**Figure 1:**
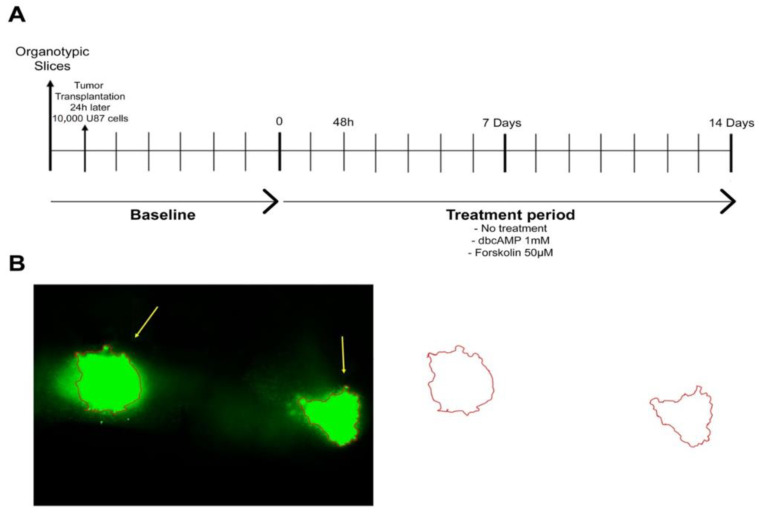
Experimental design. (**A**) Timeline. U87GFP+ cells (10,000 cells/0.1 µL medium) were implanted bilaterally into the CA3 region of the hippocampus 24 h after organotypic brain slice culture preparation. One intersection represents 24 h resp. 1 day. (**B**) Quantification of the tumor area.

On the day after OBSC preparation, 10,000 U87 cells in 0.1 µL medium were transplanted bilaterally onto the CA3 region of the hippocampus. The hippocampus is together with the subventricular zone a germinative niche in the mammalian brain, both vulnerable sites for the growth of transformed cells, because they are abundant in growth factors and permissive to proliferation [18]. The treatment started seven days after the OBSC preparation (for timeline see Figure 1A).

### 2.3. Assessment of Cell Viability with Propidium Iodide

Cell viability was assessed with propidium iodide (Invitrogen, Darmstadt, Germany) before tumor implantation and 24 h after organotypic brain slice preparation. After the application of 10 µg/mL propidium iodide for 15 min, slices were washed three times with phosphate-buffered saline (PBS), and the culture medium changed. The propidium iodide staining was repeated every week, together with washing and culture medium change. Propidium iodide is a membrane-impermeable stain that penetrates injured cell membranes and emits red fluorescence when bound to DNA. It has widely been applied for cell injury assessment. However, regaining plasmalemma integrity cells are capable of clearing propidium iodide at later times (Whalen MJ 2008; Farkas O 2006). We assessed the OBSC 2 h following propidium iodide staining and thereafter every other day.

### 2.4. Treatment of the Glioblastoma Cell Line U87 Co-Cultured with Organotypic Brain Slices

To evaluate the effect of OXPHOS augmentation, U87 GB cells cultured on organotypic brain slices were treated with the cAMP analog dibutyryl cyclic AMP (dbcAMP) at 1 mM (n = 26), and the adenylyl cyclase activator forskolin elevating cAMP at 50 µM (n = 36) added to the culture medium for 14 days. Forskolin was dissolved in 0.5% dimethyl sulfoxide (DMSO). To exclude any effect of the solvent DMSO, one tumor control group was treated with 0.5% DMSO (n = 26) and one with PBS (n = 24). Nine OBSC without tumor transplantation served as an internal control. Forty-eight hours after tumor implantation, and thereafter every week, live images were captured to document cell viability and glioma growth using an Olympus ix71microscope.

### 2.5. Biochemical Analysis

Extracellular medium samples (200 µL) were collected for measurements at the beginning of OBSC, as well as at 48 h and on days 7 and 14 following the start of treatment. Glucose, lactate, pyruvate, glutamate, and glycerol were measured with a kinetic enzymatic analyzer (CMA600, Microdialysis AB, Sweden). A standard curve was run with each set of assays using urea standards provided by the manufacturer. Samples were centrifuged and stored at −80 °C until analysis. Standard linear intervals are 2–450 mg/dL for glucose, 10–1500 µmol/L for pyruvate, 0.1–12 mmol/L for lactate, 10–1500 µmol/L for glycerol, and 1–160 µmol/L for glutamate. Each sample was analyzed in duplicate, and the mean values were averaged. To validate sample concentrations outside of the linear interval, the analysis was repeated for confirmation.

### 2.6. Quantification of Tumor Area

Images were acquired at 5× magnification with an Olympus ix71 microscope. The tumor was outlined freehand and the area was calculated with ImageJ (as demonstrated in Figure 1B). The area is given in square millimeters. All images were analyzed in triplicate.

### 2.7. Quantification of Tumor Volume by Two-Photon Fluorescence Microscopy

To assess the three-dimensional tumor volume by two-photon fluorescence microscopy, organotypic brain slices were co-cultured with the glioma cell line U87GFP+ on separate inserts with a diameter of 75 mm (Corning™ 3419, Fisher Scientific, Schwerte, Germany). Imaging was performed with a Zeiss LSM 880 NLO two-photon microscope (Zeiss, Jena, Germany) equipped with a 680–1300 nm tunable and 1040 nm fixed two-photon laser from Newport Spectra-Physics (Santa Clara, CA, USA). Two-photon images were acquired with a 20× W Plan-Apochromat objective lens (NA 1.0, Zeiss).

The glioma cell line U87 was transfected with GFP. Additionally, nuclei were stained with propidium iodide. Both fluorophores were excited at 850 nm and specific emission was detected simultaneously with non-descanned GaAsP detectors at 500–550 nm and 575–610 nm, respectively. Image stacks of the GFP channel were acquired with a z-plane spacing of 1.0 μm as tiled scans. Tiles were directly stitched in the Zeiss ZEN software.

**Figure 2:**
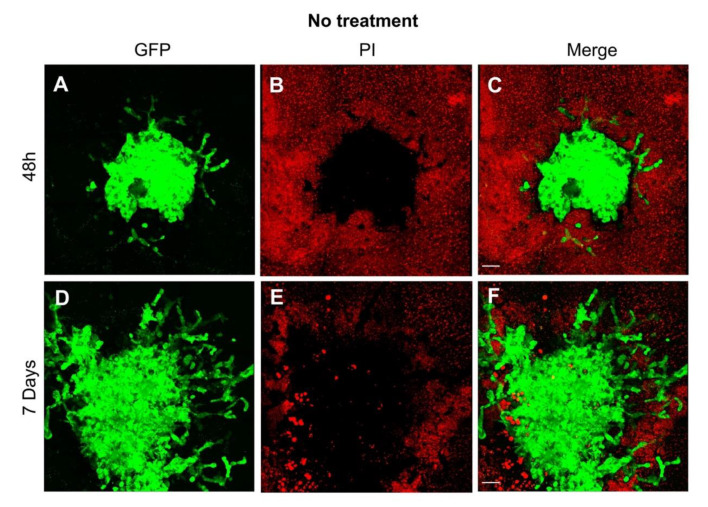
Visualization of the growth pattern of the glioblastoma cell line U87 co-cultured with organotypic brain slices. (**A**) As early as 48 h after transplantation, the tumor sphere demonstrated the first evidence of invasion as depicted by the green fluorescence of the human GB cell line U87GFP+; (**B**) the organotypic brain slices demonstrate a typical red fluorescence 2 h after propidium iodide staining; (**C**) the tumor growth on the surface of the organotypic brain slice is visualized on the merged figure; (**D**) One week after transplantation, the tumor demonstrated a considerable growth, and the GB typical invasion of the surrounding brain tissue; green fluorescence of U87GFP+; (**E**) red fluorescence 2 h after propidium iodide staining; (**F**) merged. Magnified by 20×. The scale bar represents 100 µm.

The volume of the GFP expressing tumor was quantified using Fiji [19], by manually adjusting a global threshold to separate tumor signal from the background and calculating the volume by counting foreground voxels. The data sets were visualized three-dimensionally with a 3Dscript [20]. The GFP signal is much stronger than the propidium iodide signal and bleeds into the latter channel. To make propidium iodide still visible in Figure 2, we subtracted the GFP signal from the propidium iodide channel, for illustration purposes.

### 2.8. Statistical Analysis

Investigators were blinded to experimental groups for staining, imaging, and image analysis. Initially, we performed a descriptive statistical analysis and tested for the normality of the data distribution using the Shapiro–Wilk test. Afterward, we performed a randomized two-way analysis of variance (ANOVA) for group variations followed by a Tukey post-hoc test. Statistical significance was accepted at *p* < 0.05. All analyses were performed using the Graphpad Prism software version 8 (La Jolla, CA, USA).

## 3. Results

Organotypic brain slices co-cultured with the human GB cell line U87 were utilized to demonstrate that applying cAMP activators promotes the metabolic shift from glycolysis back to OXHPOS, reduces tumor growth, and provides a differentiation therapy in GB. We analyzed different parameters in the presence and absence of dibutyryl cyclic AMP (dbcAMP, 1 mM) and the AMP activator forskolin (50 µM), such as gross metabolic effects (glucose, lactate, pyruvate, glutamate, and glycerol) in the culture medium, tumor growth in terms of area and volumetric assessment with two-photon fluorescence microscopy. To exclude any effect by the drug solvent DMSO at a concentration of 0.5%, another set of experimental control was performed with PBS. Both control groups did not differ (data not shown). Applying propidium iodide, we could confirm cell viability throughout the experimental period. The bright red fluorescence visible 2 h following propidium iodide staining faded within the next week since the cells were clearing the dye (data not shown).

### 3.1. Metabolic Effects on the Glioblastoma Cell Line U87 Co-Cultured with Organotypic Brain Slices

Xing et al. demonstrated in two GB cell lines, DBTRG-05MG and U87, that the metabolic shift from glycolysis back to OXHPOS induced by cAMP activators increased ATP level and reduced the lactate concentration [7]. The results of the biochemical analysis of glucose, lactate, pyruvate, glycerol, and glutamate in organotypic brain slices co-cultured with the GB cell line U87 and treated with the dbcAMP and forskolin are summarized in Table 1. Since the culture medium was changed every other day, samples of 200 µL were collected right before the exchange. Unlike in the cell cultures, neither a consistent effect of tumor growth nor of treatment on the metabolic situation in the culture medium (1200 µL) could be verified, most likely due to the dilution resulting in a considerable high standard deviation.

### 3.2. Reduced Tumor Area Following Differentiation Therapy

**Figure 3:**
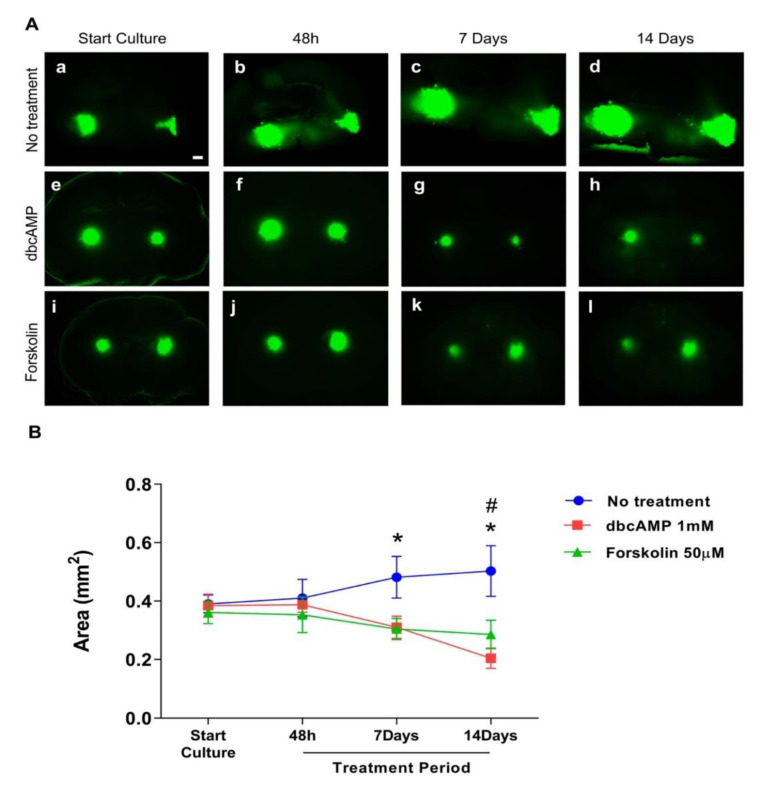
Effect of differentiation therapy on the tumor area of the glioblastoma cell line U87 co-cultured with organotypic brain slices. U87GFP+ cells transplanted bilaterally onto the CA3 region of the hippocampus after organotypic brain slice preparation. (**A**) Fluorescent images at the start of culture, and 48 h, 7 days, and 14 days after treatment (a–d untreated glioblastoma; e–h treatment with the cyclic AMP analog dibutyryl cAMP; i–l treatment with the cAMP activator forskolin; magnified by 5×. The scale bar represents 500 µm. (**B**) Graphical depiction of the results of GB area measurements with ImageJ. Group comparisons were performed by two-way ANOVA followed by a Tukey post-hoc analysis. Data are expressed as mean ± SEM (n = 8 per group). * *p* < 0.5 untreated tumor vs. forskolin; # *p* < 0.5 untreated tumor vs. dbcAMP.

The GB cell line U87GFP+ co-cultured with organotypic brain slices demonstrated without treatment the first indication of tumor growth and invasion as early as 48 h after transplantation (Figure 2A–C). One week later, the tumor volume more than doubled with an intense invasion of the surrounding brain tissue (Supplementary Video S1; 3Dview-A.mp4). Treatment with dbcAMP or forskolin resulted in a significant reduction of the tumor area over time (Figure 3, *p* = 0.0029, F_2,249_ = 5.968).

### 3.3. Visualization of 3D Growth Pattern with Two-Photon Fluorescence Microscopy

**Figure 4:**
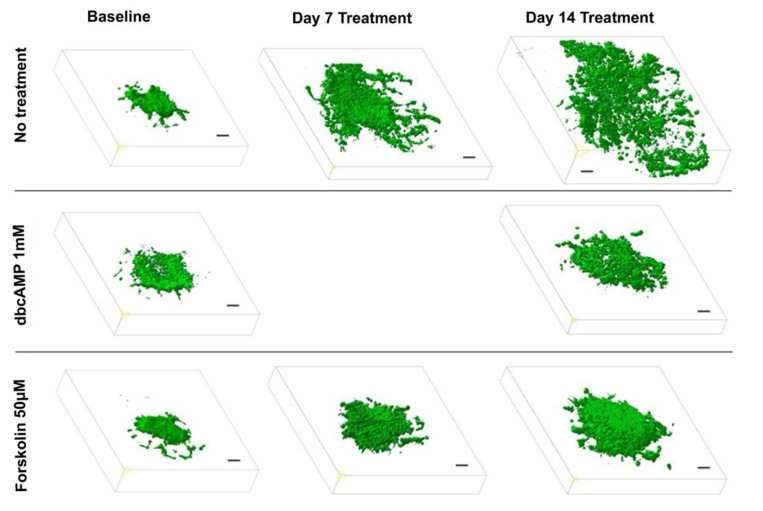
Effect of differentiation therapy on the tumor volume of the glioblastoma cell line U87 co-cultured with organotypic brain slices as quantified by two-photon fluorescence microscopy. 3D images of U87GFP+ cells transplanted bilaterally onto the CA3 region of the hippocampus before, 7, and 14 days after treatment with the cyclic AMP analog dibutyryl cAMP and the cAMP activator forskolin. Scale bars represent 100 µm.

To evaluate the effect of a differentiation therapy by OXPHOS enhancement on the GB growth pattern, we applied 2-photon fluorescence microscopy following treatment with dbcAMP (n = 6), forskolin (n = 6). Preliminary experiments demonstrated that the 6-well-dishes (diameter: 34.8 mm, 9.6 cm^2^ per well) regularly used for the OBSC-model are too small for the objective lens (diameter: 35 mm) of the 2-photon microscope. Hence, we performed the experiments with OBSC plated in larger dishes (diameter 75 mm, 44 cm^2^ per well). Since the objective lens is a water dipping lens, it has to dive into the culture medium covering the slices. This caused the floating of the slice cultures as long as they were not properly attached to the insert membrane, which took up to 10 days. Two-photon fluorescence microscopy revealed that the treated GB maintained a spheric volume pushing the brain tissue aside while the untreated controls exhibited the GB typical invasive and destructive growth pattern (Figure 4, Supplementary Video S1; 3Dview-B.mp4).

## 4. Discussion

Despite significant advances in the understanding of tumor pathogenesis, improvement in surgical techniques, and new treatment modalities, the prognosis of GB remains very poor [21,22]. In GB, a cell population with stem cell-like properties is resistant to chemotherapy. These cells exhibit specific energy metabolic characteristics [23] including reduced mitochondrial respiration as well as a preference for low oxygen concentrations to maintain their tumor-forming capacity [24]. The metabolic shift from glycolysis back to OXHPOS induced by cAMP activators, known as the “anti-Warburg effect”, resulted in two GB cell lines—DBTRG-05MG and U87—increased ATP level, reduced proliferation, and enhanced the astroglial differentiation [7]. A preliminary clinical study directly applying oxygen/ozone into the tumor vicinity of recurrent GB demonstrated a median survival rate following the first recurrence or the initiation of the treatment of 34 (range, 12–53) months [25].

To fill the gap between cell culture experiments and clinics, we utilized the well-established model of organotypic brain slices co-cultured with the GB cell line U87. We demonstrate that the treatment of GB with cAMP activators results in (1) a reduced tumor growth; and (2) a less invasive growth pattern as assessed with two-photon fluorescence microscopy.

### 4.1. Experimental Model

Organotypic brain slice culture has been adopted for neuroscience studies as a system that preserves brain architecture, cellular function, and the vascular network, and has been verified as a useful tool for the evaluation of anti-glioma drugs [15]. Tumor cells implanted close to one of the adult neural stem cell niches, the subependymal zone of the hippocampus, can effectively engraft and respond to specific signaling [14]. Furthermore, the OBSC model provides the advantage of an in vivo microenvironment thereby limiting animal experiments to the final translational phase. In contrast to extracellular matrices, OBSC allows the longitudinal 3D assessment of GB invasion and evaluation of treatment modalities (for review see [26]).

In our experimental model, we utilized OBSC from postnatal day 8 donors, as recommended because they provide optimal morphology, stable/ homogeneous susceptibility in lesion models, and increased survival for up to several months [27] as compared to adult donors [14].

However, in the case of OBSC, the slices need to be cultured for at least ten to 14 days to guarantee that they are not activated by the endogenous release of calcium or glutamate and that reactive astrogliosis is minimized. Further, developing slices need time for maturation and stabilization of intrinsic axonal projections. Only such “resting non-activated” brain slices are useful for further investigation [13].

The organotypic sections attach to the membranes a few days after being transferred to the membrane inserts and are fully attached to the membrane after two weeks in vitro [13]. This is important because the slices flatten and become transparent, which is an important macroscopic sign that the slices are healthy. The lack of thinning is the most important first macroscopic criterion of cell death or necrosis. However, to get more information on cellular viability, tissue slices must be counterstained with specific agents. Several fluorescent dyes are commercially available to directly study the viability of cells in living slices under the inverse fluorescence microscope. The most frequently used dyes are propidium iodide, as utilized in our study, ethidium bromide, SYTOX dyes, Hoechst dyes, acridine orange, DAPI, or annexin V (see for more details [28]). The advantage of this “live-cell staining” is that the slices can be investigated directly under the microscope and can be further cultured. However, all these dyes are not specific to a particular cell type and do not give information on neuronal survival [13].

The U87 cell line transplanted to OBSC in this study is a commonly studied grade IV glioma cell line that has been sequenced and analyzed in at least 1700 publications over four decades [29,30]. The GB U87 cells possess the ability of self-renewal and multipotency, and the tumors display typical histological features of human GB, including cellular pleomorphism, pseudopalisades surrounding necrosis, hyperchromatic nuclei, as well as an invasion to the brain parenchyma demonstrated in our study [31].

We evaluated U87 co-cultured with organotypic brain slices by propidium iodide staining weekly and observed that the slices were viable throughout the experimental period. Propidium iodide penetrates injured cell membranes and emits red fluorescence when bound to DNA, is one of the most common markers for membrane integrity and cell viability with specificity for damaged neurons [32], and has been applied for cell injury assessment in mixed neuronal-glial cultures [33]. However, regaining plasmalemma integrity cells are capable of clearing propidium iodide at later times [34,35]. Several applications have been reported, such as long-term live imaging [36] as performed in our study utilizing repeated two-photon fluorescence microscopy of GB growth pattern for up to 3 weeks. To demonstrate a superior contrast of OBSC and the GFP + GB cells, we acquired Figure 2 and the supplementary videos as early as 2 h following propidium iodide staining. During the following days, the bright red fluorescence was fading confirming cell viability, and was repeated thereafter weekly.

### 4.2. Metabolic Effects of Differentiation Therapy in Glioblastoma

Altered glucose metabolism in cancer cells is known as the Warburg effect, characterized by downregulated OXPHOS and upregulated glycolysis (for review [4]). Glycolysis results in the production of pyruvate, which is in the absence of oxygen further reduced to lactate. Xing et al. demonstrated in two GB cell lines that the metabolic shift from glycolysis back to OXHPOS induced by cAMP activators increased ATP level and reduced the lactate concentration [7].

The demonstration of increased extracellular pyruvate levels 2 weeks after treatment with cAMP activators would have been consistent with the hypothesis that cancer cells prefer glycolysis to generate carbon molecules for replication over energy production by OXPHOS, and that this process has been stopped at the pyruvate level [6]. The lack of a subsequent lactate increase would have indicated the capacity of respiration, thereby emphasizing that the Warburg effect serves tumor proliferation [5]. Nevertheless, given methodological problems as substrate concentrations in the 1200 µL volume of culture medium do not reliably represent an ongoing metabolic shift from glycolysis back to OXHPOS, we were not able to provide any biochemical evidence for the efficacy of the applied differentiation therapy [7]. Considering the volume of 10^6^ transplanted GB cells in comparison with the volume of a 350 mm coronal brain section, the relative contribution of metabolic tumor effects is hard to assess. We continue methodological studies applying local microdialysis [37,38].

Another metabolic substrate evaluated in our study was extracellular glutamate. It is known that glutamine metabolism is increased in cancer cells, provides ATP through glutaminolysis, and biosynthetic intermediates as glutamate for glutathione (GSH) and amino acid synthesis, malate for NADPH and pyruvate synthesis, and glutamine itself for nucleotide biosynthesis [39,40]. We expected to find extracellular changes in glutamate, but because of the mentioned methodological interference, we failed to demonstrate significant group differences.

One potential marker for cellular damage is glycerol, which is higher in patients with necrotic cerebral tumors [41]. Glycerol derives primarily from two sources in brain tissue, from the degradation of cell-membrane glycerophospholipids and glycolysis via glycerol-3-phosphate. A structural cell membrane disruption results in an increase of extracellular glycerol [42]. Applying a treatment with cAMP activators, we could not verify any difference compared to the untreated GB.

### 4.3. Effect of a Differentiation Therapy on Glioblastoma Growth and Invasion

Acting on the assumption that a metabolic shift from glycolysis back to OXHPOS, known as the “anti-Warburg effect”, may reduce the tumor-forming capacity and provide a differentiation therapy [7], we applied the cAMP analog dbcAMP and the cAMP activator forskolin to a GB model. In different brain regions, cAMP has been demonstrated to be involved in gliomagenesis. While low cAMP levels promote glioma formation, high cAMP levels inhibit the formation [7,11]. Quantifying the tumor area, we demonstrate a significant effect of the differentiation therapy with 10μM forskolin (~20% reduction) on the human GB cell line U87 [43].

To quantitatively analyze the spatial pattern of glioma cell migration and invasion of the utilized OBSC model, a 2D assessment provides insufficient data. Matsumura and colleagues introduced confocal laser scanning microscopy into the quantitative analysis of GB co-cultured with organotypic brain slices [16]. Similar to confocal laser scanning microscopy, two-photon fluorescence microscopy allows the three-dimensional visualization of the invasive GB growth pattern on the living brain slice as well as the serial analysis over several weeks. While confocal laser scanning microscopy enables a depth of penetration up to 100 µm, organotypic brain slice thickness and transplanted tumor spheres sum up to around 500 µm. Unlike confocal microscopy, excitation by two-photon enables photons to penetrate tissue with a thickness of up to 1 mm and permits the highly sensitive confocal detection of emission signals without a pinhole at the depth of up to 1 mm.

Here we demonstrate in an OBSC model, that utilizing cAMP activators reduces the tumor-forming capacity and provides a differentiation therapy in GB. Two-photon fluorescence microscopy allows a valuable quantification and visualization of tumor structure, morphology, and three-dimensional growth in our OBSC model. Specifically, we were able to demonstrate the attenuation of GB growth, invasion, and preservation of a spheric form in an organotypic model maintaining normal cytoarchitectural and biochemical properties for weeks and thereby superior to in vitro cell culture models.

## 5. Conclusions

In conclusion, we successfully facilitated an experimental model with preserved cytoarchitectural and biochemical brain properties, OBSC, for observing the effects of OXPHOS enhancement on GB growth pattern for more than 2 weeks as a proof of principle. We verified that in the presence of the cAMP activators forskolin and dbcAMP, GB proliferation was significantly inhibited. Moreover, we demonstrated that two-photon fluorescence microscopy allows the spatial and serial assessment of GB growth and invasion for several weeks. Further studies in vivo are necessary to verify the beneficial effects and to facilitate the translation into the clinical treatment.

## Figures and Tables

**Table 1:** Assessment of metabolic effects in the culture medium of the glioblastoma cell line U87 co-cultured with organotypic brain slices over time. Data are expressed as mean ± SEM (n = 8 per group) for *p* < 0.5. * difference compared to No treatment. # difference compared to No tumor. $ difference compared to Forskolin. F is the ratio of the model to its error and is calculated by dividing the mean of squares for the model and the residual mean squares.

	Baseline	Treatment with dbcAMP or Forskolin			Statistics
Extracellular Medium	U87 Trans-Plantation	Treatment	Treatment	Treatment	Two-Way ANOVA
		48 h	Day 7	Day 14	
**Glucose (mg/dL)**					F _(3,137)_ = 2.257 *p* = 0.0846
No tumor	435.89 ± 50.7	482.05 ± 49.23	550.86 ± 111.5	549.34 ± 43.16	
No treatment	506.75 ± 47.7	490.45 ± 132.9	530.66 ± 99.9	571.16 ± 79.85	
1 mM dbcAMP	480.6 ± 100.5	463.49 ± 104.9	504.95 ± 86.81	557.08 ± 99.58	
50 μM Forskolin	452.75 ± 56.2	458.04 ± 44.90	539.66 ± 68.95	502.60 ± 73.60	
**Pyruvate (μM)**					F _(3,103)_ = 3.024 *p* = 0.0331
No tumor	93.63 ± 56.23	85.37 ± 20.35	71.40 ± 19.99	53.48 ± 25.43	
No treatment	68.99 ± 32.78	53.75 ± 22.46	63.23 ± 20.99	120.23 ± 168.8	
1 mM dbcAMP	68.46 ± 39.53	80.33 ± 20.31	65.50 ± 35.70	183.5 ± 137.5 *#	
50 μM Forskolin	71.12 ± 31.87	55.37 ± 20.75	39.85 ± 24.78	147.9 ± 155.0 *#	
**Lactate (mM)**					F _(3,144)_ = 1.758 *p* = 0.1579
No tumor	6.14 ± 2.42	5.56 ± 2.48	7.15 ± 3.01	5.35 ± 1.46	
No treatment	6.45 ± 2.60	5.43 ± 2.09	5.95 ± 2.40	4.94 ± 3.90	
1 mM dbcAMP	7.33 ± 2.50	5.39 ± 2.36	4.76 ± 1.93	5.71 ± 4.07	
50 μM Forskolin	6.81 ± 3.07	4.74 ± 2.05	4.56 ± 1.55	3.01 ± 0.80	
**Glycerol (μM)**					F _(3,107)_ = 3.758 *p* = 0.0131
No tumor	217.12 ± 88.7	183.5 ± 76.15	230.86 ± 29.51	44.90 ± 63.99	
No treatment	227.3 ± 65.7	153.0 ± 73.2	145.9 ± 96.1	147.6 ± 131.0	
1 mM dbcAMP	179.6 ± 99.2	175.2 ± 107.5	226.2 ± 28.8 *	236.2 ± 154.3 $#	
50 μM Forskolin	191.1 ± 101.1	90.3 ± 77.3	75.8 ± 110.9 *	46.8 ± 67.4	
**Glutamate (μM)**					F _(3,113)_ = 0.4309 *p* = 0.7313
No tumor	123.12 ± 35.7	138.13 ± 14.90	108.71 ± 51.40	148.56 ± 24.67	
No treatment	127.6 ± 27.5	103.6 ± 44.8	106.6 ± 21.9	111.7 ± 31.0	
1 mM dbcAMP	114.9 ± 51.1	140.9 ± 36.4	132.8 ± 38.4	133.1 ± 91.2	
50 μM Forskolin	121.1 ± 47.1	134.3 ± 68.6	108.2 ± 57.5	83.0 ± 43.7	

## Data Availability

The data presented in this study are available on request from the corresponding author. The data are not publicly available since individual permission is required by the Optical Imaging Center Erlangen.

## Supplementary Materials

The following are available online at https://www.mdpi.com/2073-4409/10/3/556/s1, Video S1: The GB cell line U87GFP+ co-cultured with organotypic brain slices demonstrated without treatment tumor growth and invasion as quantified by two-photon fluorescence microscopy, 3Dview-A.mp4. Video S2: Following treatment, i.e., reversing the Warburg effect, the tumor keeps the original size and spheric shape without any signs of invasion, 3Dview-B.mp4.
